# Control of disseminated intravascular coagulation in Klippel-Trenaunay-Weber syndrome using enoxaparin and recombinant activated factor VIIa: a case report

**DOI:** 10.1186/1752-1947-4-92

**Published:** 2010-03-19

**Authors:** Ulf H Beier, Mary Lou Schmidt, Howard Hast, Susan Kecskes, Leonard A Valentino

**Affiliations:** 1 Division of Pediatric Hematology and Oncology, Department of Pediatrics, University of Illinois at Chicago, Chicago, Illinois, USA; 2 Pediatric Critical Care Medicine, Department of Pediatrics, University of Illinois at Chicago, Chicago, Illinois, USA; 3 The RUSH Hemophilia and Thrombophilia Center, Department of Pediatrics, Rush Children's Hospital and Rush University Medical Center, Chicago, Illinois, USA

## Abstract

**Introduction:**

Vascular malformation is associated with coagulopathies, especially when hemostasis is challenged.

**Case presentation:**

We present the case of an 11-year-old Hispanic girl with Klippel-Trenaunay-Weber syndrome that developed disseminated intravascular coagulation after minor surgery, which was controlled by blood product transfusions and enoxaparin to address an ongoing consumptive coagulopathy. The patient, however, developed bacteremia and liver trauma that resulted in severe bleeding. To the best of our knowledge, we report here the first known instance of administering recombinant coagulation factor VIIa to control acute bleeding in a patient with Klippel-Trenaunay-Weber syndrome.

**Conclusions:**

This case illustrates the concept of enoxaparin maintenance to suppress an ongoing consumptive coagulopathy and the use of recombinant coagulation factor VIIa to control its potentially fatal severe bleeding episodes.

## Introduction

Vascular malformations are associated with coagulopathies, especially in patients challenged by the stress of surgery [1,2]. A major problem previously associated with the condition is inaccurate diagnostic classification, as many of these vascular abnormalities require different methods of treatment [3,4]. There are two principal categories of vascular abnormalities associated with bleeding. One category includes those which arise secondary to cellular proliferation like the Kasabach-Merritt syndrome (KMS) with predominant platelet trapping, while the other includes those in which the etiology is based upon the distortion of the vascular bed, which leads to continuous activation and subsequent consumption of clotting factors [4,5].

Meanwhile, the Klippel-Trenaunay syndrome is a triad of port-wine stains, varicose veins, and osseous or soft tissue hypertrophy involving one or multiple extremities. When combined with arteriovenous malformations, the syndrome develops into the Klippel-Trenaunay-Weber syndrome (KTWS), a condition that can be associated with both KMS and consumptive coagulopathy [6,7].

## Case presentation

An 11-year-old Hispanic girl with previously diagnosed KTWS and numerous vascular abnormalities in her lower extremities was transferred from an outside hospital three days after the resection of a valvular capillary hemangioma. A physical examination and a computed tomography (CT) scan confirmed the presence of KTWS by showing diffuse hemangiomas, varicosities, and arteriovenous malformations involving the soft tissues of the pelvis and the bilateral lower extremities that had caused bilateral lower limb hypertrophy, mostly on the right limb.

There was massive postoperative bleeding from the wound with an estimated blood loss of 14.7 ml/kg in the first five hours after the procedure. Disseminated intravascular coagulation (DIC) ensued with increased D-dimers and low levels of fibrinogen (95 mg/dl). The patient was transfused with 15 ml/kg of packed red blood cells one day after the operation. Hemostasis was eventually achieved at 13 days after the operation. However, due to ongoing DIC, daily transfusions with packed red blood cells, platelets, cryoprecipitate, and fresh frozen plasma were required.

**Figure 1:**
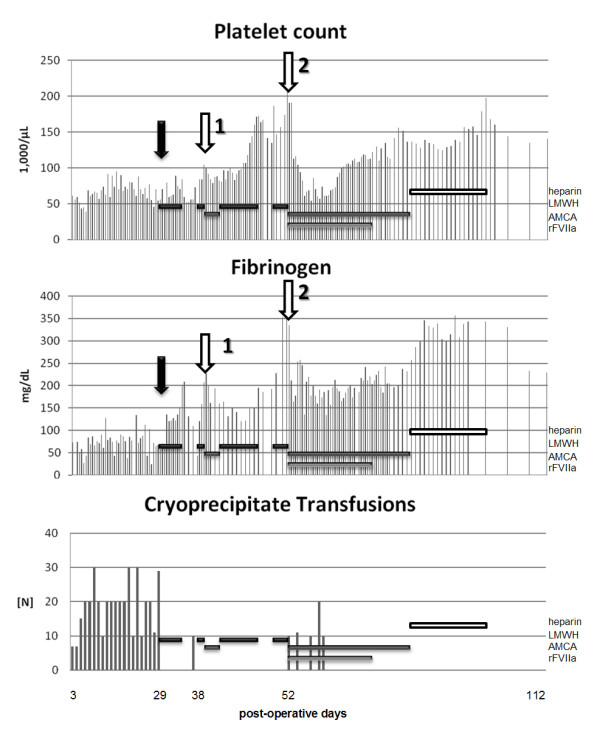
**Postoperative platelet counts, fibrinogen levels, and cryoprecipitate transfusions**. The black arrow indicates initiation of low-molecular-weight heparin (black bars). The white arrow #1 indicates the onset of bacteremia and the white arrow #2 indicates cholecystomy drainage placement with subsequent liver laceration. The interval of aminocarpoic acid administration is indicated by the dark grey bars. The initiation (arrow #2) and duration of administration of recombinant activated factor VII is indicated by a light grey bar, while the duration of unfractionated heparin administration is indicated by a white bar.

Following the criteria set by Mazoyer *et al. *[4], we considered a condition of consumptive coagulopathy secondary to venous malformations. A therapy of low-molecular-weight heparin (LMWH) (enoxaparin, 1 mg/kg once daily) was therefore begun on day 29. The bleeding abated abruptly, hence transfusions of blood products were no longer needed (Figure 1). The bleeding was controlled until the patient became febrile and bacteremic at 38 days after the operation.

Triggered by bacteremia with *Citrobacter youngae *and *Enterococcus faecalis*, DIC recurred and bleeding resumed 40 days after the operation with up to 53 ml/kg of blood replacements per day. Enoxaparin was discontinued and aminocaproic acid (100 mg/kg) was administered to limit fibrinolysis. The bleeding subsided on the following day. When the acute bleeding stopped, the patient's blood count improved under resumed enoxaparin therapy and the patient was no longer in need of blood product transfusion until day 52.

Meanwhile, the patient had developed cholestasis that required cholecystostomy drainage on the 53^rd ^day following the initial hemangioma surgery. Two days later, the patient's hemoglobin dropped precipitously and massive ascites developed. This led to elevation of the patient's diaphragm and respiratory distress that resulted in the need for intubation. An exploratory laparotomy showed a subcapsular tear and hematoma. Enoxaparin was suspended while numerous transfusions, aminocaproic acid and, for the first time, recombinant activated factor VII (rFVIIa, NovoSeven^®^, Novo Nordisk, Denmark, 30 μg/kg every 2 hours) were administered to the patient. The rFVIIa was continued for 12 days, then gradually tapered over an additional 15 days. Subsequently, the bleeding originating from the hepatic laceration subsided.

The patient was discontinued on rFVIIa on day 79 and aminocaproic acid on day 88. While enoxaparin maintenance therapy was reconsidered, it was not initiated because of its long half-life and the risk of recurrent bleeding due to liver laceration. Unfractionated heparin (70 units/kg subcutaneous once daily) was given while the patient was immobilized. She recovered and was discharged on day 112. Enoxaparin (1 mg/kg) was restarted and continued for six months after hospital discharge. At the one year follow-up, evidence of ongoing localized intravascular coagulation persisted with elevated D-dimer (6.4 to 5.5 μg/ml) and reduced fibrinogen levels (116 to 227 mg/dl). The patient is still on enoxaparin (1 mg/kg) as her only medication, attending school including a physical education class (with the limitation of contact sports), ambulating, and receiving physical therapy.

## Discussion

The case presented demonstrates several important points regarding the treatment of postoperative bleeding in KTWS. Despite postoperative complications and the recurrence of varicosities, vascular surgery remains an indispensable component of KTWS treatment considering the amount of pain and the hemostatic complications that could arise if not corrected [8]. While conducting surgery, efforts were placed upon the avoidance of bleeding complications. Terada *et al. *successfully applied endoscopic sclerotherapy with mono-ethanolamine oleate to prevent bleeding [9]. In a recent review of KTS, Gloviczki and Driscoll emphasized the importance of proper imaging prior to the procedure, using intraoperative tourniquet to decrease the risk of bleeding, and the importance of a multidisciplinary approach in addition to intraoperative hemostatic procedures [10].

Once bleeding complications occur, identification of the pathophysiologic mechanism leading to the hemostasis imbalance is critical, that is, whether there is a platelet pooling like in KMS as opposed to continuous local intravascular coagulation that consumes coagulation factors, leading to the formation of thrombin and fibrin within anatomical structures that either slow down or distort the blood stream.

While KMS is known to respond to steroids and antiproliferative agents [11], these agents have no known effect in venous malformation associated with consumptive coagulopathy [4]. In our patient, hemostasis was best achieved by preventing localized thrombosis via elastic stockings and LMWH. However, these were only effective in the absence of other potent prothrombotic stimuli, such as sepsis or trauma. Our patient, when challenged by infection and liver laceration, developed DIC despite enoxaparin therapy, thus requiring aggressive replacement of cellular and soluble blood components to maintain hemostasis. As enoxaparin alone failed to prevent DIC, aminocaproic acid and rFVII were administered to temporarily shift the balance from an anticoagulant to a procoagulant milieu.

Patients with KTWS have been occasionally administered with antifibrinolytic agents. Poon *et al. *administered aminocaproic acid to a KTWS patient with vascular malformations and a platelet sequestration syndrome [12]. Katarsos *et al. *gave tranexamic acid successfully to an adult patient with KTWS who was also undergoing severe visceral bleedings [13]. However, the use of rFVIIa has not previously been reported in the treatment of KTWS. We hypothesize that rFVIIa was successful in controlling bleeding in our patient because baseline localized intravascular coagulation progressed to DIC, thus depleting the coagulation factors. LMWH could not prevent the consumption of the coagulation factors, which is a pathologic feature of KTWS [4]. Although the chief hematological problem of KTWS is a procoagulative state [14], once consumption is too extensive, it converts to an anticoagulant state [15,16]. Once coagulation was again achieved and the underlying challenge was resolved, we found resuming heparin effective in controlling baseline prothrombotic tendencies induced by the vascular malformations.

## Conclusions

Although heparin, along with physical therapy measures, is the mainstay of KTWS maintenance therapy, it may prove insufficient in cases when severe DIC has developed. In these situations, after supplementing coagulation factors and cellular blood components, hemostasis may be achieved with the administration of rFVIIa. Furthermore, we suggest that KTWS vascular surgery, if indicated, be done under a multidisciplinary approach that is able to respond to complications that could arise secondary to trauma. However, rFVIIa, aminocaproic acid and LMWH could all lead to life-threatening thrombotic complications. Close monitoring by a highly skilled multidisciplinary team is thus necessary.

## Abbreviations

CT: computed tomography; DIC: disseminated intravascular coagulation; KMS: Kasabach-Merritt syndrome; KTWS: Klippel-Trenaunay-Weber syndrome; LMWH: low-molecular-weight heparin; rFVIIa: recombinant activated factor VIIa.

## Consent

Written informed consent was obtained from the parents of the patient for publication of this case report and any accompanying images. A copy of the written consent is available for review by the Editor-in-Chief of this journal.

## Competing interests

The authors declare that they have no competing interests.

## Authors' contributions

UHB wrote the manuscript and compiled the figures. LAV and MLS edited the manuscript. All authors analyzed and interpreted the patient data regarding the hematological disease and its management. All authors read and approved the final manuscript.
